# A review of complex *in vitro* cell culture stressing the importance of fluid flow and illustrated by organ on a chip liver models

**DOI:** 10.3389/ftox.2023.1170193

**Published:** 2023-04-24

**Authors:** John Malcolm Wilkinson

**Affiliations:** Department of Materials Science and Engineering, Sheffield University, Sheffield, United Kingdom

**Keywords:** microphysiological systems, organoids, microfluidics, millifluidics, physiological relevance, DMPK, perfusion, animal replacement

## Abstract

The translation of new technology from development into widespread commercial use is a complex and time-consuming process that requires significant investment. This review looks at some important market needs for more complex *in vitro* models, the technical difficulties that must be overcome, particularly those connected with introducing fluid flow using microfluidics, and also illustrates the economic benefits of more accurate models for drug toxicity. Beyond the strong ethical arguments for replacing the use of animals in drug safety testing and medical research, the author believes that financial benefits of adopting the new *in vitro* technology are becoming clear and will drive the adoption by industry.

## 1 Introduction

It is widely accepted that the pharmaceutical industry needs a more physiologically relevant *in vitro* model to deliver better predictivity of drug toxicity and efficacy. Over 10 years ago, there was great excitement about the emergence of a new technology which has become known as ‘Organ on a Chip’ (OOAC) because it was believed that OOAC might meet this need. There was a surge in development activity and investment by government agencies and venture capital. Most organisations developing OOAC chose to use microfluidics devices made using microfabrication technology which has a long history of success in the electronics industry because it has delivered increased functionality, better performance and economic benefits in microelectronic chips. However the physics of liquid flow in biological systems is very different from that controlling electrons in a microelectronic circuit. After 10 years of intense development, it is worth looking at the status of OOAC technology and assessing what is needed to deliver the transformation that was expected.

It is disappointing that advanced *in vitro* methods including OOAC have as yet been unable to deliver even a small reduction in the use of animals for the assessment of drug safety. There seem to be at least three contributing factors: i) lack of understanding about the conflicting market requirements between animal replacement in medical research and the needs of High Throughput Screening (HTS) in drug development ii) the technical difficulties associated with the development of more complex models, especially in use of microfluidics and iii) the need for evidence of a strong economic advantage to industry before the adoption of any new technology by industry.

This paper addresses each of these factors in turn. It has focused on hepatotoxicity but it is clear that the ability of OOAC to address other frequent toxicities causing failures in clinical trials, such as cardiotoxicity and immunotoxicity, will need to be demonstrated to support more widespread adoption. Once more accurate predictions of toxicity can be made then the next challenge will be testing efficacy of drugs which is also a major reason for drug failure.

A major justification for the use of animals in testing is the need to look at the effects of drugs on multiple and interconnected organs. For OOAC technology to be commercially successful in replacing the use of animals it will need to demonstrate its ability to model multiple connected organs. This paper significantly updates the conclusions of an earlier review (Wilkinson (2019)).

## 2 Application requirements and market needs

### 2.1 Overview

The pharmaceutical industry uses a combination of *in vitro* tests and animal experiments to screen new drug compounds prior to clinical trials. *In vitro* models have traditionally been single organ models but are evolving towards more complex multi-organ systems. Single-organ tests allow evaluation of the response of a specific organ to a compound or mixture of compounds. Multi-organ systems aim to evaluate the potential interaction of one organ with at least one other, principally through the exchange of metabolites or soluble signaling molecules. Single organ models are most commonly a single cell type in a 96 or 384 well plate. The more complex models are not in standard well plates but customized designs that are now being referred to as microphysiological systems. They typically incorporate multiple cell types from a single organ (e.g., hepatocytes, stellate cells and Kupffer cells for the liver model), As well as the shift to multiple cell types, the complexity is being increased by more tissue like cultures (3D) and perfusion of medium rather than static (zero flow). The introduction of flow can stimulate cell viability and function as well as providing the mechanism to connect multiple chambers The choice of a single-organ or multi-organ system depends on the desired functionality needed for the system to be a good model of the physiological processes.

**FIGURE 1:**
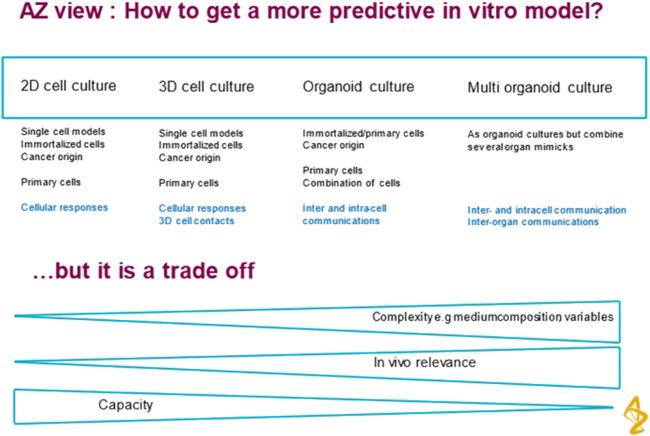
Road map 2D to 3D to Flow to organoid to multi-organ. The author is grateful to Astrazeneca for permission to reproduce Figure 1.

The degree of complexity should be kept to the minimum required to represent the biological application without introducing unnecessary factors that make the system difficult to use and analyse. Figure 1 shows the trade-offs involved. In addition to the trend towards more complex models, there has been a focus of interest in miniaturization using microfluidic technology. However it is not essential to miniaturise to achieve physiological relevance and predictive models. This paper explores the idea that there may be an optimal scale for such models that is closer to millimeter size. The motivation to miniaturise is driven by past success in microelectronics where smaller transistors operate more quickly, use less power and are more economic to manufacture in volume. These benefits do not translate to biological devices. High Throughput Screening has moved from 96 to 384 well plate format and although this has increased throughput, it has not delivered more predictive assays.

A further design consideration is the choice of the cell material to use in the *in vitro* device. This can range from using primary tissue (e.g., an organ slice from a biopsy) or bottom up growth of tissue into small organoids. These can be grown from cells from primary, immortalized lines or stem cells. Cells of human origin are preferred over animal sources. There is also a growing interest in animal free serum and animal derived extra cellular matrix.

The conflicting technical demands between building an animal replacement technology and delivering more predictive High Throughput Screening (HTS) are explored in the following sections Even though animals are complex biological system models, they do not mimic human metabolism.

### 2.2 *In Vitro* models for HTS

HTS assays are designed to give a fast go/no go decision on the safety of a drug compound before it is progressed to the very expensive later stages of drug development. An assessment of toxicity needs to be sensitive (must catch most toxic compounds) and specific (avoid giving false positives or false negatives) It is accepted that a more predictive *in vitro* model will undoubtedly require an increase in complexity over the current HTS *in vitro* assays. This will involve compromises and trade-offs between accuracy and throughput which were elaborated in a recent presentation from AstraZeneca (see Figure 1). HTS also tends to focus on one factor of toxicity rather than exploring multiple modes of action. This simplifies the end points to be detected. A further discussion is given in Section 3.4.

Toxicity can be assessed by measuring the dose of the potential drug that will kill 50% of the cell population in the culture plate This concentration is known as the IC50 value. For assessment of liver toxicity, it is now recognized that cells in culture must not only be viable for the duration of the dosing regimen but must express the phase I and phase II metabolizing enzymes as well as uptake and efflux transporters. Many simple liver cell culture assays have failed this criterion because even though freshly harvested human liver cells are active, within 3–7 days most of their metabolic activity has declined and they do not correctly metabolise the drugs to which they are exposed.

Adding medium flow and moving to 3D tissue like cultures can improve liver cell viability and function for as long as 28 days (Vinci et al., 2011) but the resulting models and protocols are more complex.

The strategy of many OOAC developers is to try to develop automated machines that will hide the complexity of the protocols and operational details from the users. The justification for this approach is that most existing HTS assays are highly automated with complex and expensive robotic systems but they do deliver massive amounts of data that feeds in to the candidate selection process.

### 2.3 Animal replacement

Animals are used in industry (Pharma and Contract Research Organisations) in parallel with HTS assays as a further way to assess the risk of toxicity as well as being used in academic research on disease mechanisms. In the former application, the requirements for sensitivity and specificity are the same as they are for HTS assays.

Later stages of drug development use animals as a way to assess dosing strategies. Drug Metabolism and Pharmacokinetics (DMPK) help analyse how quickly a drug is metabolized and excreted as well as indicating where drugs accumulate in other organs. Building *in vitro* models of these processes is far more complex than developing HTS assays. Not only must cell cultures be viable for longer periods but transport of drugs and metabolites between multiple organ models is needed.

A different application is the use of animals in Academic Research for studies on disease. The technical requirements for a replacement method are similar to DMPK but the economics and strength of the ethical arguments are worth contrasting with the industrial applications.

Despite the high cost of using animals and ethical arguments, the justification for their use is that multi-organ models are required to detect toxicity and study complex diseases. The animal is, after all, a fully functioning systemic model of metabolism. Technical arguments that animal metabolism is different from human biology have so far been insufficient to convince regulators that change is necessary. The pharmaceutical industry has distanced itself from the ethical tensions by relying on Contract Research Organisations (CRO’s) to carry out the animal work. So in order to replace animals in drug safety assessment both the Pharma client and the CRO service deliverer have to be convinced of the efficacy and accuracy of any alternative even before the Regulators get involved. An alternative is for Government and Regulators to force change by banning the use of animal testing, as they did with its use in the testing of Cosmetics.

Reducing the use of animals in academic research may be an easier challenge to solve than changing industry. Medical researchers in academia may be more sensitive and responsive to the ethical arguments. In addition they may have sufficient understanding of disease mechanisms that they can define a multi-organ model that does not need the complexity of the whole body. For example, a liver/pancreas/heart model might be sufficient even if still technically very challenging. It is projected that an incremental growth in *in vitro* models may allow the complexity to gradually build as new individual organs/organoids are added. This strategy seems to be part of the TissUse GmbH approach. (Ramme et al., 2018).

The economic analysis of these two distinct market opportunities is discussed in the next section.

## 3 Economic analysis

### 3.1 Potential benefits of using OOAC to displace HTS

The important corollary to Figure 1 is that although *in vivo* relevance is a desirable criterion, it is the commercial benefit that will drive acceptance of more complex models by industry. This can only come from a significant improvement in the success rate of new drug candidates in clinical trials. It seems the pharma companies are putting the onus on the OOAC technology companies to validate their new methods by testing a portfolio of compounds with known positives and negatives. Hence several of the companies (Emulate, CNBio, Hesperos) have adopted a CRO model to develop and pay for the necessary expertise.

Proving the efficacy of the OOAC technology is now the main focus of at least one of the development companies (Ewart et al., 2022) In a recent study, Emulate analysed the ability of their commercial Liver Chip model to predict Drug Induced Liver Injury (DILI) caused by small molecules. An economic analysis was also performed to measure the value Liver-Chips could offer if they were broadly adopted in supporting toxicity-related decisions as part of preclinical development workflows. The main impact would be to cut out drugs that are going to fail in clinical trials earlier. This would save many times the additional costs of using Liver Chips. To estimate the economic impact of incorporating the Liver-Chip into preclinical research, Emulate observed that DILI currently accounts for 13% of clinical trial failures that are due to safety concerns. Their study revealed that the Liver-Chip would have an ability to catch 87% of compounds that evaded traditional safety workflows. Combining these figures suggested that adding the human Liver-Chip to existing workflows to test for DILI risk could lead to 11.3% fewer toxic drugs entering clinical trials.

It remains to be seen whether these arguments are accepted by the pharmaceutical industry in general. It will clearly be more convincing when equivalent data is available on the ability of OOAC to predict the other frequent toxicities that lead to drug failure in clinical trials (e.g., cardiotoxicity and immunotoxicity).

### 3.2 Economics of replacing animals

In section 2.1 it is suggested that a strong economic argument will be the strongest justification for industry to adopt new methods and this would also apply to the take-up of non-animal methods. To assess the current situation, it is important to look at the costs of animal tests and how they compare with the capital and consumable costs of the *in vitro* alternatives. There is an excellent analysis of Animal Costs by Bottini and Hartung. (2009). Their 2005 data gives a total cost of animal tests in Europe of €620million for 1.026 million animals or an average cost of €604 per animal. Some tests are far more expensive and use large numbers of animals. Sub chronic toxicity with over 11 thousand animals at $3,884 each and long-term repeated toxicity using 37 thousand animals at $2,499 each illustrate the expense of running long term multi-animal trials.

Most toxicity assessments need at least 4 different doses and triplicates of each experiment. Hence a ballpark figure for a standard animal test using rodents would be 12 animals at a cost of $330 each (this includes all breeding, growth, test and disposal). So the target cost that a non-animal *in vitro* assay has to match is $3,960 per compound or drug.

It is difficult to get equipment costs from many OOAC developers as they have adopted a CRO model and are not selling their OOAC plates or equipment very widely. It appears that the capital cost of the control system and pumps is between $50,000 and $100,000. This represents a significant investment by any academic research laboratory wishing to adopt the technology, although it may well be less of a challenge for industrial or CRO labs. The individual single use OOAC chips are priced between $250 and $400.

Whereas commercial pharmaceutical companies frequently use CRO service providers, this approach is of little interest to academic users who need novelty in the way they configure *in vitro* models even if the building blocks of these models are standard components.

The high prices for OOAC chips and control equipment from commercial companies is a further incentive for academic laboratories to build their own version of OOAC technology. This is clear from the large number of published research papers on new OOAC designs and has led to a proliferation of different architectures. This makes results difficult to compare and hinders the more widespread adoption of the technology. In addition many of the academic OOAC chips are fabricated in polydimethylsiloxane (PDMS), a material that suffers from high absorption of many biological materials and small molecule drugs (see Section 4.4).

The supply of standard building blocks to academic users is the basis of a different business model from the company, Kirkstall Ltd. The control and pumping system costs from Kirkstall are as much as 10 x lower than some competing OOAC systems. The consumable plates offer 6 or more culture chambers in one plate that sells in the same price range as some single experiment OOAC chips.

An alternative approach is being piloted by Emulate Inc. This company has installed its equipment to Queen Mary College at the University of London and is offering access to other academic users in exchange for a fee. (Knight, 2023).

## 4 The technical difficulties of complex *in vitro* models

### 4.1 Physics of flow

The physics of flow can explain why microfluidics, which uses narrow flow channels, creates problems which are not familiar to researchers working on conventional static cell cultures. Phenomena such as bubble formation, surface tension, evaporation and nutrient depletion, are far more difficult to solve at the microscale volumes and material choices of many OOAC’s. It becomes far more important to model mass transport phenomena controlled by viscosity and diffusion to see how they influence the supply of oxygen and nutrient and removal of waste from the cells. Under laminar flow conditions, resistance to flow through a fine channel is inversely proportional to the fourth power of the tube radius r^4^ (Poiseuille’s law) so a small capillary tube of diameter 200um will have a resistance per unit length 625 times higher than a tube of 1 mm diameter.

As *in vitro* model complexity increases, it would be beneficial for cell biologists and those designing OOAC devices to have a much deeper understanding of the physics governing their complex models. Building the complexity can be an incremental process. The first step is to stay with a single cell type but move to a 3D or more tissue like culture. 3D cultures can be grown on a supporting scaffold or extra cellular matrix (ECM). It is interesting that cells under perfusion flow will often grow their own ECM so the next step is to add perfusion of medium. This has the added benefit that a reservoir bottle of medium can be in the flow circuit and that can enable longer term cultures without the need for frequent medium changes. It is also the enabling step for the next level of complexity: the move to connected multi chamber models. At this stage more cell types from the target organ can be added to recapitulate the full metabolism of the target organ. The final step is to approach a full systemic model with multiple organoids communicating. At this level there is a chance that the functionality could be enough to replace a full animal model.

The first technology that must be mastered is fluid flow. This is important, even for single cell type 3D models which will become necrotic in the centre if thicker than 300um in a static medium culture. Cell culture biologists can benefit from understanding the physical properties that influence flow (Surface tension, viscosity, flow stress) and the effect that flow has on cells. Epithelial junctions are tighter under flow (Luissint et al. (2012)) whereas hepatocyte viability can be damaged (Mazzei et al., 2010). Flow supplies oxygen to cells and removes waste products. It can also subtly influence metabolism in the liver particularly if there are gradients of pO2 pressure. (Tomlinson et al. (2019))

Fluid flow in microfluidic channels is particularly difficult to optimize and leads to failure of many OOAC devices. It was Michael Shuler, currently President of Hesperos, who first pointed out that air bubbles are a serious obstacle to a successful operation of a long-term microfluidic systems using cell culture (Sung and Shuler (2009)). However another difficulty is the lack of medium flow and low oxygen delivered to the cells. There are two simple approaches to solving this problem: the first is to simply increase the radius r of the flow channels, and the second is to increase the pressure. Several commercial OOAC developers have taken the latter approach (Emulate, CNBio and TissUse). Unfortunately, this dramatically increases the capital cost and complexity of the control system for the OOAC device.

A different strategy has been adopted by Kirkstall. The chamber and tubing diameters have been increased to millifluidic scale so that low pressures are enough to create adequate flow. Simple peristaltic pumps can then be used. These pumps are low cost and can fit inside a standard cell culture incubator, which is not the case for high pressure pneumatic pumps. The result is a technology which is affordable and yet flexible enough to build multi-organ models.

### 4.2 Pressures used in organ on a chip

For Organ on a Chip devices, the goal should be to limit the pressures that the cells experience to those found in the human body and certainly below those found in large Arteries (systolic 120 mm of Hg to diastolic 80 mm of Hg) These pressures should then guide the selection of pumping mechanisms for use in Organ on a Chip devices. If higher pressures are used then there is a risk of rupturing the cell walls of the tissue under culture.

Syringe pumps use a mechanically driven actuator and are a low-cost solution where single pass flow is required. However they must be used with care because if a blockage develops in the flow circuit the pressure will rise and values as high as 3 x atmospheric pressure have been reported (3bar or 3000 mm of Hg).

In early Organ on a Chip Research it was hoped that silicon chip based micropumps manufactured by photolithography and using the piezoelectric effect would be effective way to pump medium round flow circuits. However, micropumps were ineffective because they could not overcome the high resistance of microfluidic channels. Many of the OOAC developers who originally planned to use micropumps have now migrated to externally driven pneumatic pumps using compressed air or laboratory vacuum lines switched through miniature valves. These valves can be on the chip (for example, TissUse) or on the cartridge that holds the chip (for example, CNBio).

Pneumatic pumps can also deliver dangerously high pressure (2 x atmospheric pressure or 2000 mm of Hg) unless there is a pressure monitor in the flow circuit.

Peristaltic pumps are more limited in the pressures they can support at around 0.1bar to 0.2 bar (100mm–200 mm of Hg) and these are well within the normal physiological range.

Although not strictly a pump, Rocker Plates are also used to induce flow under gravity and the equivalent pressure is 0.001–0.01 atmospheric (1–10 mm of Hg). If this type of gravity driven flow device is connected to microfluidic channels with high resistance the levels of flow will be very limited.

### 4.3 Recirculating *versus* single pass flow

A recirculating flow has the advantage of recirculating signalling molecules, which allows chemical communication between the different elements of a multi-organ device. Recirculation, however, does not allow for continuous replenishment of nutrients, and will lead to an accumulation of waste products. The length of time that an OOAC systems can be operated without medium exchange depends on the ratio of medium volume to number of cells in the device, and can vary from daily exchange to weekly removal of a portion of medium from the OOAC with replacement by fresh medium [Mazzei et al.]. Medium removal has the useful benefit that material is provided for offline analysis. The flow conditions are also important for maintaining the oxygen supply to the cells. Computer simulation can be extremely helpful in the design of chambers and flow systems Hyndman et al. (2020).

### 4.4 Adsorption of molecules to plastic materials

A further consideration in the choice of which material to use for fabrication of cell culture systems is that PDMS or other elastomeric polymers can adsorb small hydrophobic molecules [Toepke and Beebe (2006) ] This is particularly important in microfluidic circuits, where the surface area to volume ratio is high. Surface adsorption can lead to nutrient or ligand depletion so giving rise to experimental artifacts such as increased metabolic consumption rates. These effects have been modelled with a variety of materials and chamber geometries Pedersen et al. (2016).

Where *in vitro* assays are used to define toxic doses for hazard characterization, the concentration–effect relationships are frequently based on nominal concentrations, i.e., the amount of chemical added to the system divided by the volume of the exposure medium. However, because of absorption effects, the nominal concentration is not necessarily the concentration reaching cells or target sites where toxic events are initiated. This is particularly important for DMPK and mode of action studies, where the nominal concentration does not necessarily represent the exposure concentration responsible for the observed effect. Surfactants accumulate at interphases and are likely to sorb to *in vitro* system components such as serum protein and well plate plastic. Groothuis et al. (2019).

Absorption of chemicals in plastic and, if present, proteins must be considered along with other assay setup conditions such as cell density, repeated dosing and exposure time, all of which can influence the observed toxic potency as well.

To improve the predictivity of *in vitro* assays for unwanted effects of drugs, the distribution of compounds in the *in vitro* system, between the cells, the microtitre plate plastic and medium needs to be measured over time. Kramer et al. (2015) This paper showed that lipophilic drugs bind significantly to plastic labware. A few drugs, including less lipophilic drugs, bind to cell-attachment matrices.

### 4.5 End point analysis

During culture, simple monitoring of cell viability can be useful and simple visual inspection is adequate. At the end of the assay, whereas HTS relies on a very simple end point and short culture time, OOAC and devices hoping to replace animals are more complex. Although simple assessments of toxicology may be helpful, in many cases more information on DMPK and disease mechanisms is needed. Medium samples can give information on cell status, gene and protein analyses. In other cases, access to the cell culture will be essential. This is another issue for many microfluidic devices which may need the device be dismantled to perform end point histology, lytic assays, transcriptomics and even cell viability assays.

Ideally OOAC designs would allow microscopy and high-content imaging. as these are among the most widespread analytical methods in cell biology.

### 4.6 Making complex biological models physiologically relevant

**FIGURE 2:**
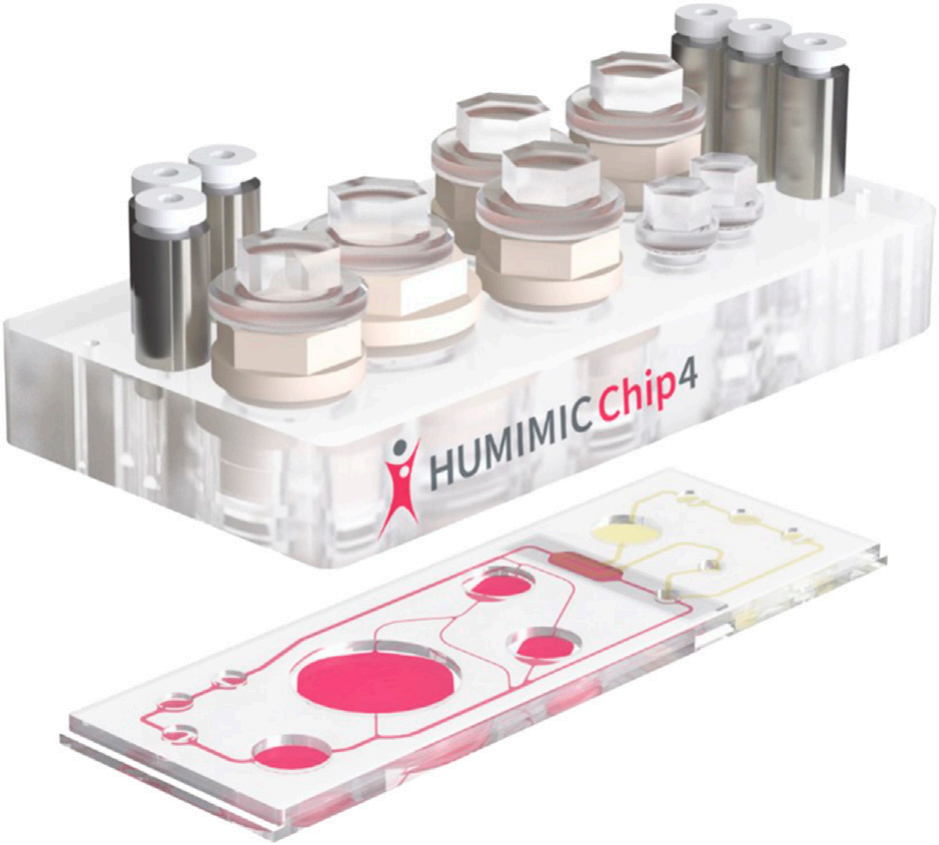
HUMIMIC Chip exploded view. The author is grateful to TissUse GmbH for permission to use Figure 2 under License: CC BY-ND 4.0.

Since the start of OOAC development over 10 years ago, many single organ/organoid devices have been developed and reviewed in the literature (Leung et al. (2022)). However, single organ *in vitro* models are not adequate to meet the needs for animal replacement. Although the initial vision of OOAC in 2012 was that more than 10 organoid models would be connected together, this had not been achieved by 2022. Some OOAC developers appear to be pursuing a single organ on a chip plan (Ewart et al. (2022)) and this has already demonstrated improved performance over HTS and simple organoid assays. In contrast, TissUse GmbH have successfully demonstrated a four organ co-culture by 2018 (Ramme et al., 2018) with intestine, liver, neuronal and renal cells. Figure 2 shows the schematic of the TissUse HUMIMIC chip.

The HUMIMIC Chip is formed of a number of chambers (similar in size to a 24 well plate chamber) formed in plastic sitting on top of a base plate that connects the chambers by microfluidic channels. The largest chamber is a fluid reservoir. At the extreme left and right in Figure 2 are seen the triplets of connections which link to the external pumping equipment to move liquid media between the cell culture chambers and the fluid reservoir. Easy access to the reservoir or cell culture chambers is provided by screw on caps. These allow cells to be seeded into the chambers, media to be changed if necessary in a long term culture or cells/media to be extracted for analysis.

The criteria for a systemic *in vitro* model to be physiologically relevant are far more stringent than a single cell model, It was highlighted in Section 2 that the criteria for a single cell *in vitro* model to replace HTS is a significant improvement in the sensitivity and selectivity of the toxicity assays as was demonstrated by Ewart. Drug toxicity assessment depends on accurate assessment of IC50 values for a candidate drug compound. The cell culture conditions must enable the cells to express the correct CYP P450 enzymes Although freshly harvested human hepatocytes do show high CYP activity, this can degrade within two or 3 days in a static culture. Introduction of correct flow levels can restore the CYP activity (Vinci et al., 2011).

**FIGURE 3:**
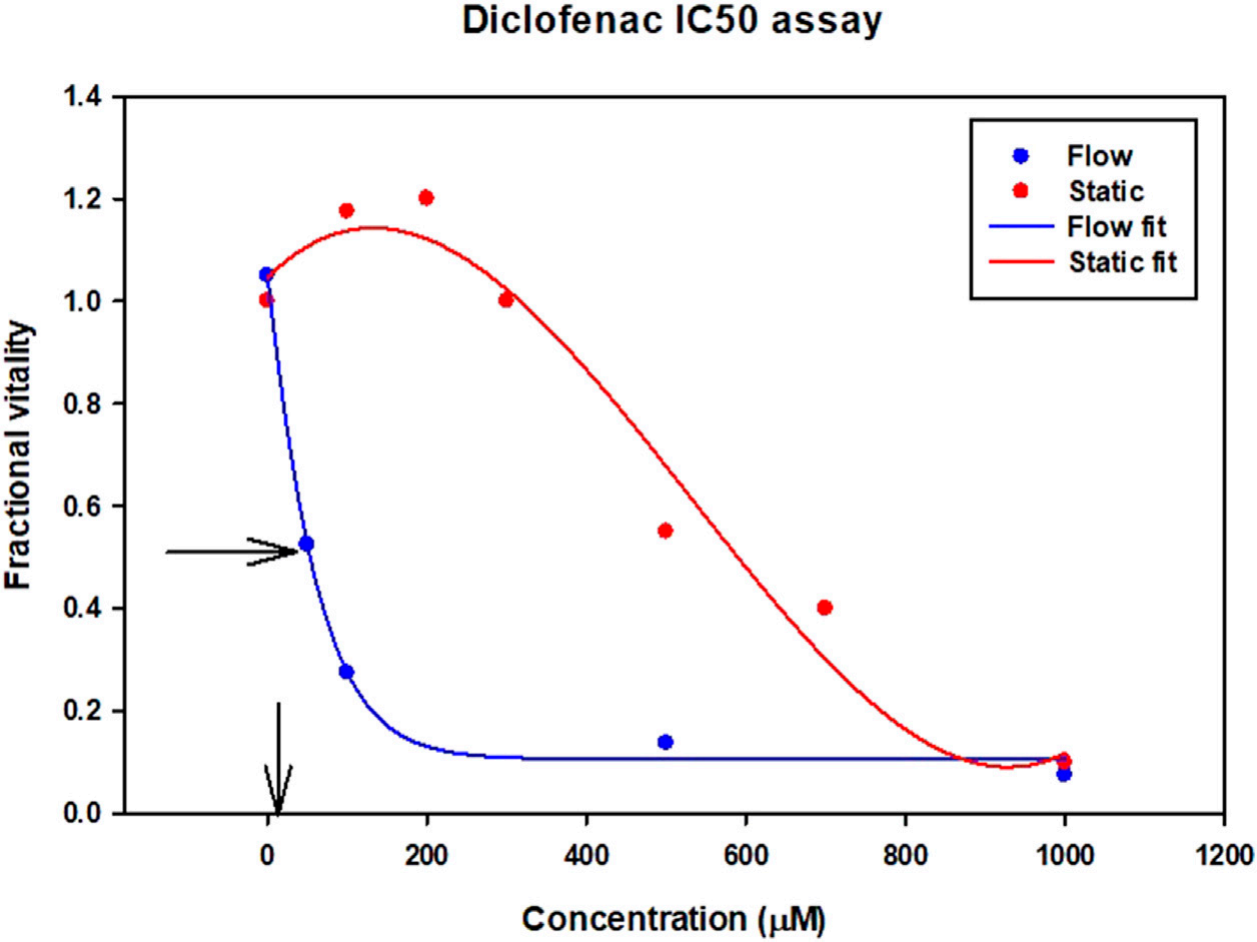
Diclofenac Drug Toxicity illustrated by IC50 results for different assays. Vertical arrow on x axis indicates clinical IC50 value from clinical trials.

The expression of CYP enzymes and transporters at the correct (physiological) level is particularly important for toxicity assessment of drugs like diclofenac. Diclofenac itself is not particularly toxic compared to its metabolites. IC50 is the drug concentration that kills 50% of the cells. In Figure 3 although the clinical data suggest the toxicity IC50 value is 4.2 uM (shown by the arrow on the x axis), the data from a static cell culture (zero flow) suggests that 140 times higher dose of diclofenac can be tolerated. Introducing flow (which stimulates metabolism) gives a ten times closer fit to the clinical data.

## 5 Comparison of some representative OOAC products

**FIGURE 4:**
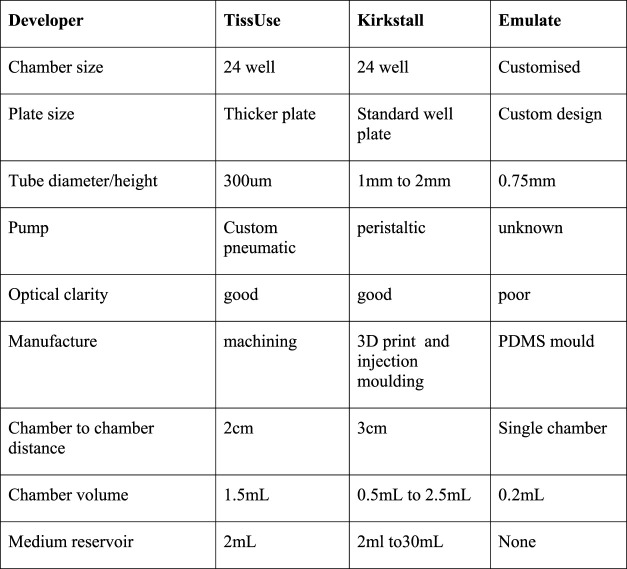
Comparison of TissUse GmbH, and Kirkstall Ltd., and Emulate Inc. OOAC features.

There are an enormous number of OOAC devices under development, many in university research groups, some in small start up companies and a somewhat smaller number in revenue generating commercial companies. This review focuses on the technologies and products of three companies: Emulate Inc. United States of America, TissUse GmbH. Germany, and Kirkstall Ltd. United Kingdom. Figure 4 shows a comparison of the technical features of the OOAC devices from TissUse GmbH, Kirkstall Ltd., and Emulate Inc.

The technology, protocols and results from the Emulate LiverChip have recently been published (Ewart et al. (2022)) TissUse GmbH appears to the furthest ahead with multi-organ models (Ramme et al. (2018)) and Kirkstall Ltd., has the largest user base in Academic Laboratories (Singh et al. (2021))

**FIGURE 5:**
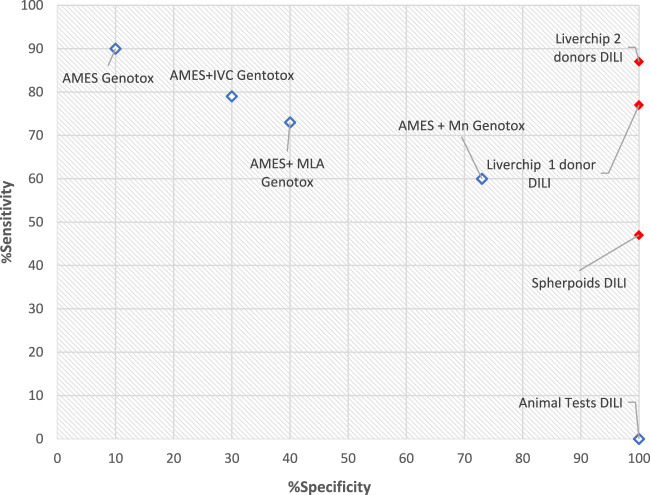
Sensitivity vs. Specificity for Assays.

The Emulate work summarized the results of tests on 870 individual human liver chips to determine how well they predicted drug induced liver injury across a set of 27 known toxic and non-toxic drugs. The Emulate Liver Chip outperformed conventional *in vitro* models (including the latest spheroid cultures) and gave an 87% chance of predicting liver toxicity compared to 47% with spheroid 3D models and 0% with animal tests. Figure 5 compares the recent results for Liverchip with cells from one and two donors as well as the results from Spheroids by plotting sensitivity against specificicty. The plot also includes some commercially available genotoxicity assay results for comparison. The high specificity but low sensitivity of animal tests explains why they fail to identify drugs which then proceed to clinical trials.

Although Ewarts paper makes a convincing economic case for investing in OOAC development it suggests that OOAC is not a replacement but is an adjunct to current animal and HTS assays. Figure 5 shows the Emulate DILI toxicity results in the context of other widely used assays for a single toxicity hazard, in this case genotoxicity assays. As previously stated, an ideal assay will have a specificity of 100% (to avoid false positives) and a sensitivity of 100% (to avoid false negatives).

The Emulate paper suggests the following next steps for development of their Liver Chip.

i) Need to change from PDMS to some other plastic with non-specific binding
ii) Need to check model validity for more than just Drug Induced Liver Injury (DILI) as this only accounts for 13% of drug compound attrition in Clinical Trials
iii) Model must be tested with large molecule drugs

Neither Kirkstall or TissUse have carried out large scale testing of single organ models at the level completed by Emulate. Both are focusing on the development of multi-organ systemic capability.

## 6 Route from single organ OOAC to systemic models

The goal is to move from validated and robust single organ OOAC devices to multi-organ devices that mimic the systemic physiology of the human body. These will not only be used to evaluate the safety and efficacy of pharmaceutical compounds but will become more important tools for medical research on chronic human diseases in general and an important step on the way to replacing the use of animals. Models of metabolic crosstalk and the interactions between metabolically active organs, such as skeletal muscle, the liver, adipose tissue, the pancreas and the gut, will be needed to study diseases such as type 2 diabetes and non-alcoholic fatty liver disease, which are growing global health concerns.

Tools aimed at the academic research community will need to demonstrate the following.

i) Affordability
ii) Ease of use
iii) Community of Practice/Technical Support

**FIGURE 6:**
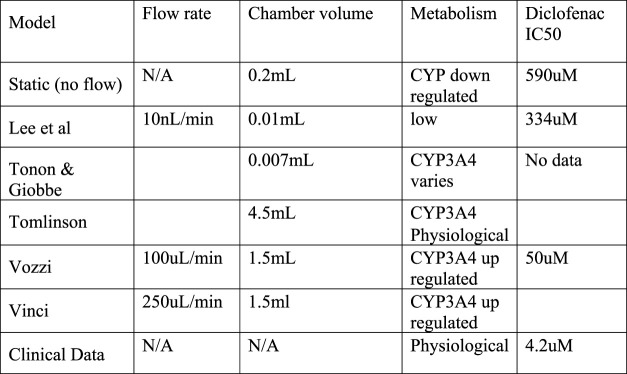
IC50 values for Liver models.

Both the TissUse HUMIMIC systems and the Kirkstall Quasi Vivo System are candidates that are well on the way to meeting these criteria. Figure 6 compares some of the technical features of these two offers against the Emulate device. One of the important differences is the tubing diameter between chambers. Since the flow resistance is inversely dependent on the fourth power of the tube diameter for a given length of tube the TissUse device has123 times more resistance to flow between chambers than the Quasi Vivo device. If the cells need a specific flow to provide oxygen then the pressure provided by the TissUse pump must be 123 times higher than the Kirkstall Quasi Vivo pump. This illustrates why TissUse have selected pneumatic pumping rather than peristaltic.

## 7 Conclusion

Although advanced *in vitro* methods including OOAC have not yet delivered a significant reduction in the use of animals for the assessment of drug safety, the prognosis is that the potential has clearly been demonstrated. The factors which have slowed the translation of the technology into the market are being addressed. The conflicting market requirements between animal replacement in medical research and the needs of High Throughput Screening (HTS) in drug development are more clearly understood.

Further reduction in the use of animal models will require.

• Additional evidence that OOAC offers better sensitivity and specificity than animal tests in other areas of toxicity than just hepatotoxicity
• Evidence that drug efficacy can be better demonstrated in OOAC than animal models
• Emergence of standards both in equipment architecture and operating protocols for OOAC
• Emergence of multi-organ or all major organs in vitro models

The business models and market focus of the companies developing OOAC are now evolving to meet the differing requirements. The technical difficulties associated with the development of more complex models, especially in use of microfluidics are better understood and being solved. The first evidence for the economic advantages available from the adoption of the new technology by industry and academia is emerging.
