# Supplementary material for: A review of complex in vitro cell culture stressing the importance of fluid flow and illustrated by organ on a chip liver models
Source: Front Toxicol. 2023 Apr 24;5:1170193. doi: 10.3389/ftox.2023.1170193 (PMC10165094; doi:10.3389/ftox.2023.1170193)
Supplement: Supplementary file 1 [file Table1.DOCX]

Figure 1 Tradeoffs in complexity for in vitro models. Figure courtesy of AstraZeneca


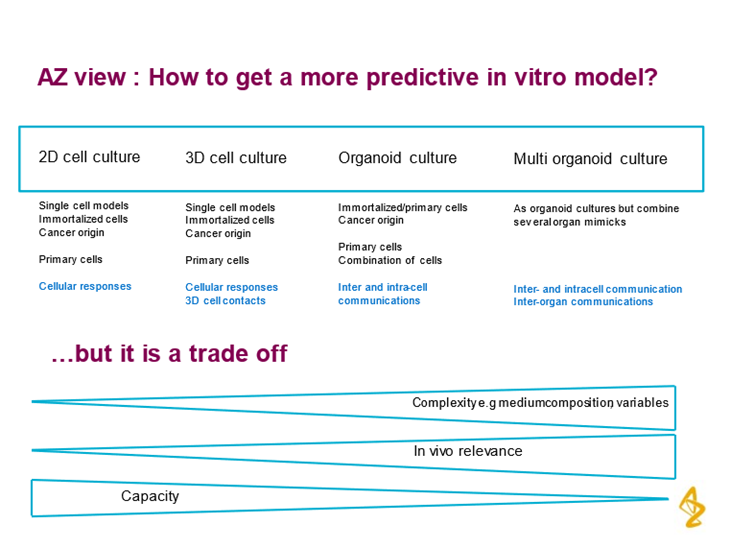


Figure 2 HUMIMIC Chip exploded view (Author: TissUse GmbH, License: CC BY-ND 4.0).


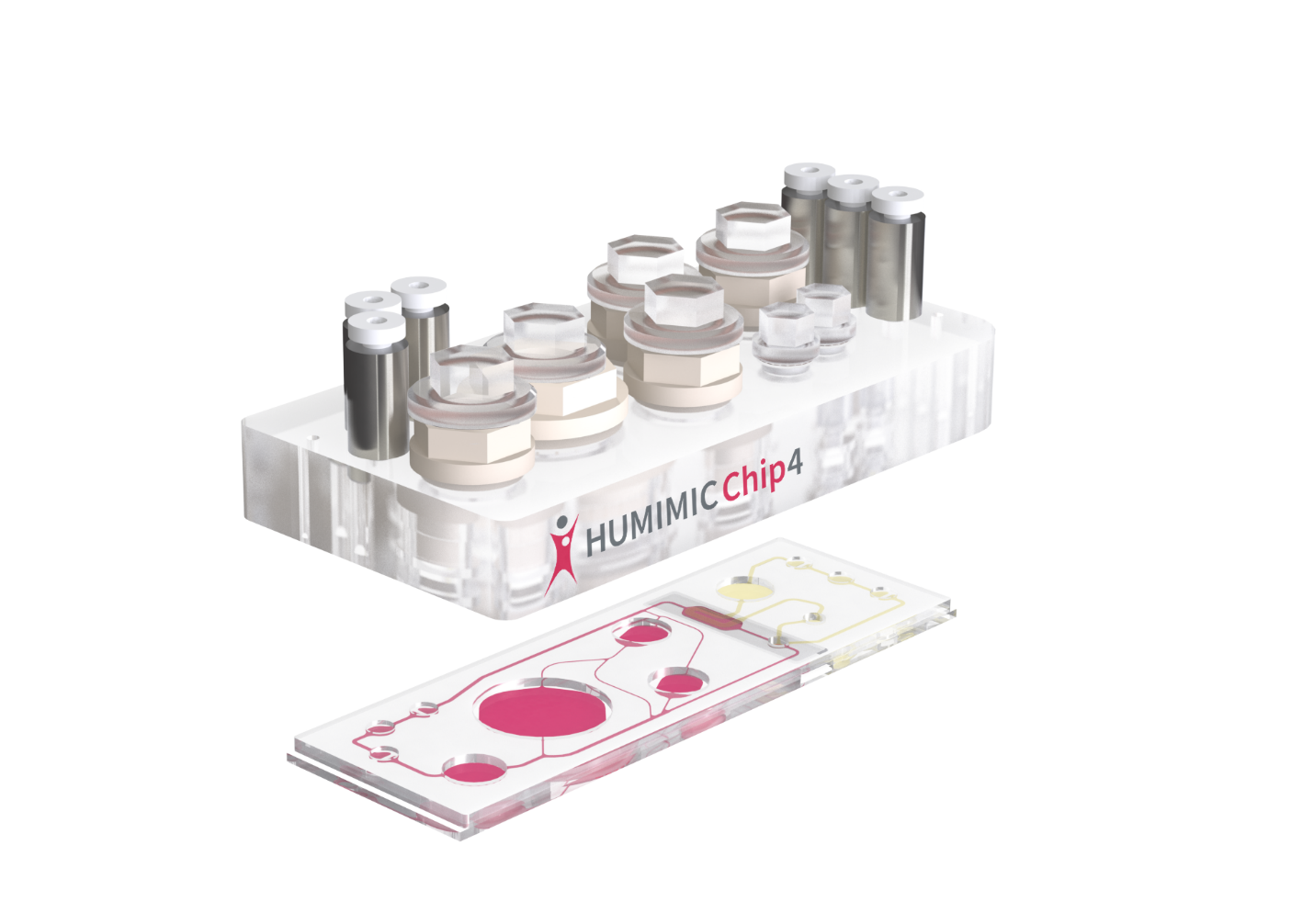


Figure 3 Diclofenac Drug Toxicity illustrated by IC50 results for different assays


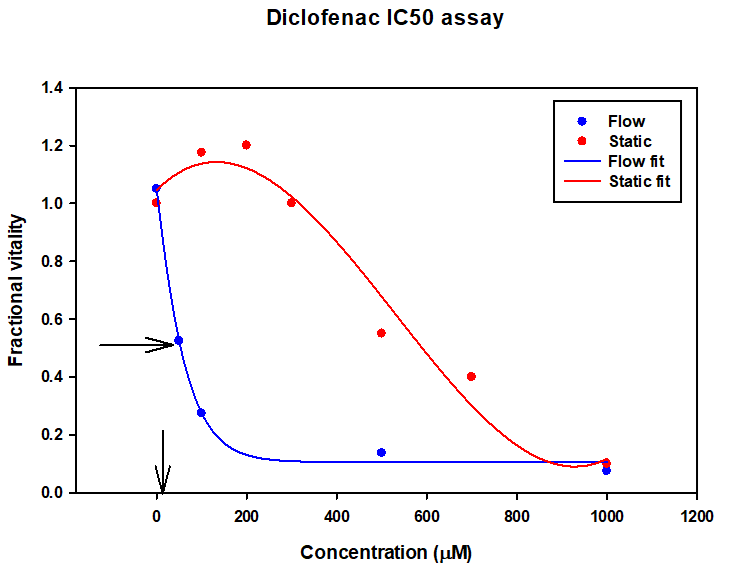


Figure 4 IC50 values for Liver models

| - Model | - Flow rate | - Chamber volume | - Metabolism | - Diclofenac IC50 |
| --- | --- | --- | --- | --- |
| - Static (no flow) | - N/A | - 0.2mL | - CYP down regulated | - 590uM |
| - Lee et al | - 10nL/min | - 0.01mL | - low | - 334uM |
| - Tonon & Giobbe |  | - 0.007mL | - CYP3A4 varies | - No data |
| - Tomlinson |  | - 4.5mL | - CYP3A4 - Physiological |  |
| - Vozzi | - 100uL/min | - 1.5mL | - CYP3A4 up regulated | - 50uM |
| - Vinci | - 250uL/min | - 1.5ml | - CYP3A4 up regulated |  |
| - Clinical Data | - N/A | - N/A | - Physiological | - 4.2uM |

Figure 5 Comparison of TissUse GmbH, and Kirkstall Ltd., and Emulate Inc. OOAC Features

| **Developer** | **TissUse** | **Kirkstall** | **Emulate** |
| --- | --- | --- | --- |
| Chamber size | 24 well | 24 well | Customised |
| Plate size | Thicker plate | Standard well plate | Custom design |
| Tube diameter/height | 300um | 1mm to 2mm | 0.75mm |
| Pump | Custom pneumatic | peristaltic | unknown |
| Optical clarity | good | good | poor |
| Manufacture | machining | 3D print and injection moulding | PDMS mould |
| Chamber to chamber distance | 2cm | 3cm | Single chamber |
| Chamber volume | 1.5mL | 0.5mL to 2.5mL | 0.02mL |
| Medium reservoir | 2mL | 2ml to30mL | None |

Figure 6 Sensitivity vs Specificity for Assays
